# Larotrectinib versus Prior Therapies in Tropomyosin Receptor Kinase Fusion Cancer: An Intra-Patient Comparative Analysis

**DOI:** 10.3390/cancers12113246

**Published:** 2020-11-04

**Authors:** Antoine Italiano, Shivani Nanda, Andrew Briggs, Jesus Garcia-Foncillas, Ulrik Lassen, Gilles Vassal, Shivaani Kummar, Cornelis M. van Tilburg, David S. Hong, Theodore W. Laetsch, Karen Keating, John A. Reeves, Marc Fellous, Barrett H. Childs, Alexander Drilon, David M. Hyman

**Affiliations:** 1 Early Phase Trials Unit, Institut Bergonie, 33000 Bordeaux, France; 2 University of Bordeaux, 33076 Bordeaux, France; 3 Bayer HealthCare Pharmaceuticals, Inc., Whippany, NJ 07981, USA; shivanin@hmplglobal.com (S.N.); karen.keating@bayer.com (K.K.); john.reeves.ext@bayer.com (J.A.R.); marc.fellous@bayer.com (M.F.); barry.childs@bayer.com (B.H.C.); 4 London School of Hygiene & Tropical Medicine, Bloomsbury, London WC1E 7HT, UK; Andrew.Briggs@lshtm.ac.uk; 5 OncoHealth Institute, University Hospital Fundación Jiménez Díaz, 28040 Madrid, Spain; jgfoncillas@quironsalud.es; 6 Department of Oncology, Rigshospitalet, 2100 Copenhagen, Denmark; ulrik.lassen@regionh.dk; 7 Institut Gustave Roussy, 94800 Villejuif Cedex, France; Gilles.Vassal@gustaveroussy.fr; 8 Stanford Cancer Institute, Stanford University, Palo Alto, CA 94304, USA; kummar@ohsu.edu; 9 Hopp Children’s Cancer Center Heidelberg (KiTZ), Heidelberg University Hospital and German Cancer Research Center (DKFZ), 69120 Heidelberg, Germany; cornelis.vantilburg@kitz-heidelberg.de; 10 Investigational Cancer Therapeutics, University of Texas MD Anderson Cancer Center, Houston, TX 77030, USA; dshong@mdanderson.org; 11 Department of Pediatrics and Harold C, Simmons Comprehensive Cancer Center, University of Texas Southwestern Medical Center/Children’s Health, Dallas, TX 75390, USA; LAETSCHT@chop.edu; 12 Memorial Sloan Kettering Cancer Center, New York, NY 10065, USA; drilona@mskcc.org (A.D.); dhyman@loxooncology.com (D.M.H.); 13 Weill Cornell Medical College, New York, NY 10065, USA

**Keywords:** growth modulation index, larotrectinib, *NTRK* gene fusion, tropomyosin receptor kinase, TRK fusion cancer

## Abstract

**Simple Summary:**

Clinical trials for new drugs to treat rare diseases are difficult to evaluate due to the limited patient population available for recruitment. Growth modulation index (GMI) is a very useful tool in these instances, as this calculation compares the patient’s outcome on the current drug to the same patient’s outcome on their most recent prior therapy, using the patient as their own control. GMI is the ratio of progression-free survival on the current therapy to time to progression on the last prior line of therapy and offers a method to determine if the investigational drug provides a benefit compared to the patient’s last prior treatment. Using a GMI ≥ 1.33 as the threshold of meaningful clinical activity, we found that larotrectinib, a tropomyosin receptor kinase (TRK) inhibitor approved to treat patients with TRK fusion cancer, improves progression-free survival for most patients with TRK fusion cancer compared with prior therapy.

**Abstract:**

Randomized controlled basket trials investigating drugs targeting a rare molecular alteration are challenging. Using patients as their own control overcomes some of these challenges. Growth modulation index (GMI) is the ratio of progression-free survival (PFS) on the current therapy to time to progression (TTP) on the last prior line of therapy; GMI ≥ 1.33 is considered a threshold of meaningful clinical activity. In a retrospective, exploratory analysis among patients with advanced tropomyosin receptor kinase (TRK) fusion cancer treated with the selective TRK inhibitor larotrectinib who received ≥1 prior line of therapy for locally advanced/metastatic disease, we determined the proportion of patients with GMI ≥ 1.33; patients who had not progressed by data cut-off were censored for PFS. Among 72 eligible patients, median GMI was 2.68 (range 0.01–48.75). Forty-seven patients (65%) had GMI ≥ 1.33; 13/25 patients (52%) with GMI < 1.33 had not yet progressed on larotrectinib. Kaplan–Meier estimates showed a median GMI of 6.46. The probability of attaining GMI ≥ 1.33 was 0.75 (95% confidence interval (CI), 0.65–0.85). Median TTP on previous treatment was 3.0 months (95% CI, 2.6–4.4). Median PFS on larotrectinib was not estimable ((NE); 95% CI, NE; hazard ratio, 0.220 (95% CI, 0.146–0.332)). This analysis suggests larotrectinib improves PFS for patients with TRK fusion cancer compared with prior therapy.

## 1. Introduction

Neurotrophic tyrosine receptor kinase (*NTRK*) gene fusions are oncogenic drivers in a wide variety of tumors [1]. Larotrectinib is a specific tropomyosin receptor kinase (TRK) inhibitor that is approved by the US Food and Drug Administration and European Medicines Agency (EMA) to treat patients with TRK fusion cancer [2,3]. In three phase I/II clinical trials (adult phase I, NCT02122913; SCOUT, NCT02637687; NAVIGATE, NCT02576431) evaluating the efficacy and safety of larotrectinib in patients with TRK fusion cancer, larotrectinib demonstrated tumor-agnostic efficacy (objective response rate (ORR), 79%) and a favorable safety profile in patients with TRK fusion cancer, irrespective of age, the *NTRK* gene, or fusion partner [4,5].

Single-arm studies are common for cancers with rare oncogenic drivers due to the low number of patients available for recruitment. However, they do not provide comparative data. Studies of experimental anticancer therapies have traditionally used tumor response outcomes as endpoints, but measures of tumor progression may be more relevant in the study of novel targeted therapies [6]. While progression-free survival (PFS) is a widely-used measure of tumor growth delay, it can demonstrate high inter-trial variability due to patient-level prognostic factors even after correction for these factors [7]. The growth modulation index (GMI) is an innovative measure that can be implemented in single-arm studies, defined as the ratio of their PFS on the current therapy to the time to progression (TTP) on the most recent prior line of therapy for individual patients; therefore, patients serve as their own control. Since TTP typically decreases with each subsequent line of anticancer therapy, a GMI of >1 would suggest that an investigational therapy is having a positive impact on a tumor’s natural history. A GMI of ≥1.33 indicates a ≥33% improvement in PFS over the previous line of therapy and has been proposed as a threshold of meaningful clinical activity [8]. The EMA has endorsed the use of GMI as an endpoint for truly rare tumors or very narrow indications [9] and several phase II studies of cytotoxic anticancer treatments have implemented this approach to compare activity with prior treatment [10,11,12,13,14,15]. Despite the original proposal of incorporating GMI analysis into the evaluation of cytostatic therapies, this analysis has been used to enable comparison between multiple therapeutic regimens for a given individual.

Here, we use GMI to evaluate the intra-patient impact of larotrectinib compared with previous systemic therapies in patients with TRK fusion cancer.

## 2. Results

At the time of data cut-off, 72 patients were eligible for analysis, including 53 patients (74%) with metastatic disease. In the overall dataset of 72 patients, 36 patients (50%) were male. There were 21 pediatric patients (29%) aged < 18 years; 10 (14%) were <5 years old. Overall, 63 patients (88%) had an Eastern Cooperative Oncology Group (ECOG) performance status of 0 or 1. Twelve tumor types were included in the analysis, with soft tissue sarcoma (*n* = 26 (36%)) and salivary gland (*n* = 9 (13%)) being the most common. Forty-six patients (64%) had received ≥2 prior therapies. TRK fusions involved *NTRK1* (49%), *NTRK2* (3%), and *NTRK3* (49%). Of the 53 patients with metastatic disease, 22 (42%) were male and 11 (21%) were pediatric with one patient (2%) < 5 years old. The two most common tumor types in patients with metastatic disease were soft tissue sarcoma (*n* = 15; 29%) and lung cancer (*n* = 7; 13%). The other baseline characteristics were well balanced with those of the overall analysis set (Table 1).

**Figure 1:**
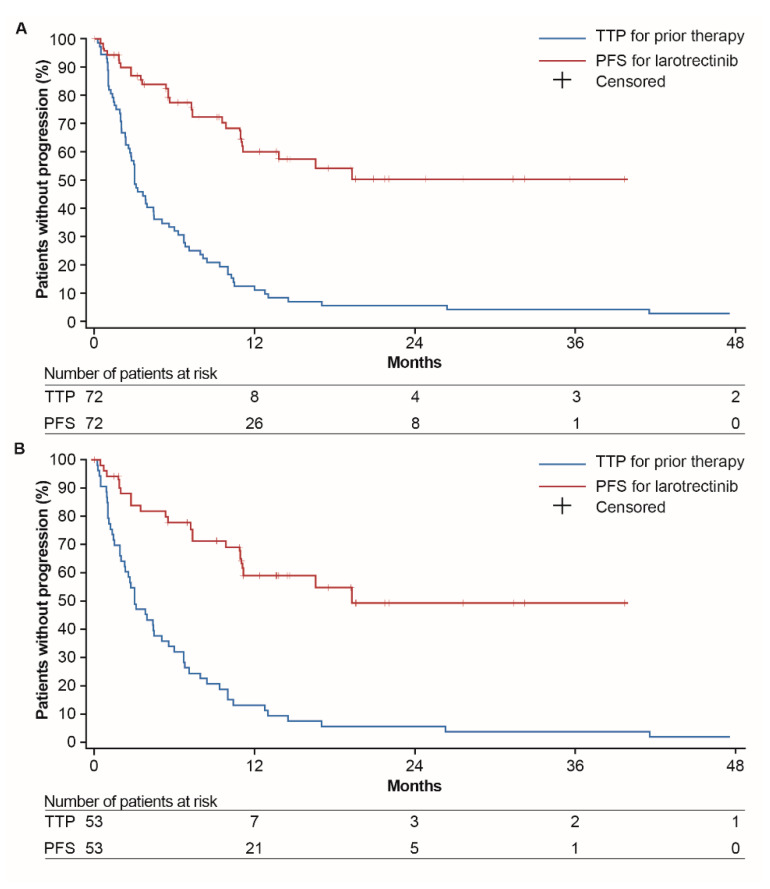
Kaplan–Meier plot of progression-free survival on larotrectinib (per IRC) and time to progression on the previous line of therapy (per investigator assessment) for (**A**) the overall dataset (locally advanced or metastatic disease (*n* = 72)) and (**B**) patients with metastatic disease (*n* = 53). For TTP, one patient was censored at 122 months and one patient had progressed at 151 months. Abbreviations: IRC, independent review committee; PFS, progression-free survival; TTP, time to progression.

For all patients (*n* = 72), median TTP on the previous line of treatment was 3.0 months (95% CI, 2.6–4.4) and median PFS on larotrectinib was not estimable (NE) (95% CI, NE; hazard ratio (HR), 0.220 (95% CI, 0.146–0.332); Figure 1A). The confidence interval reflects the clustering within matched pairs (TTP and PFS) by using a robust variance estimator [16]. A total of 45 patients were censored for PFS. Among patients with metastatic disease (*n* = 53), median TTP on the prior line of treatment was 4.4 months (95% CI, 2.8–6.1) and median PFS on larotrectinib was 19.3 months (95% CI, 10.9–NE; HR, 0.228 (95% CI, 0.146–0.357); Figure 1B).

**Figure 2:**
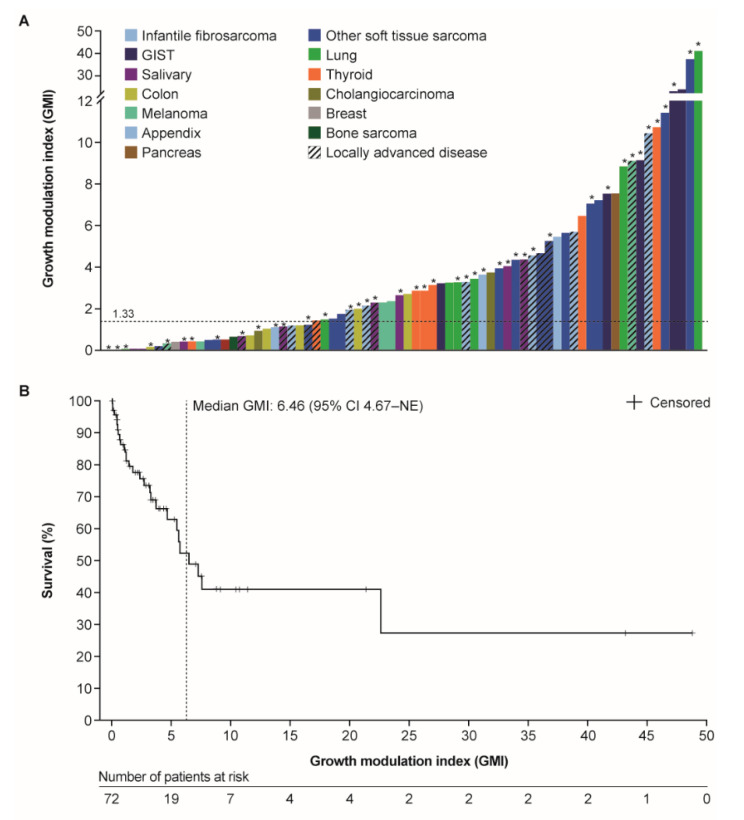
(**A**) Waterfall plot of individual patient growth modulation indices for the overall cohort (locally advanced or metastatic disease (*n* = 72)) by tumor type; (**B**) Kaplan–Meier estimate of median GMI. GMI was defined as the ratio of progression-free survival on larotrectinib (independent review committee assessment) to the time to progression (investigator assessment) on the most recent prior line of therapy. * Patient had not progressed at the time of data cut-off. Abbreviations: CI, confidence interval; GIST, gastrointestinal stromal tumor; GMI, growth modulation index; NE, not estimable.

In the overall dataset and utilizing individual patient PFS on larotrectinib and prior TTP on last therapy, the arithmetic median GMI was 2.68 (range 0.01–48.75). In total, 47 patients (65%) had a GMI of ≥1.33, 42 patients (58%) had a GMI of ≥2, and 19 patients (26%) had a GMI of ≥5. There were 25 patients (35%) with a GMI of <1.33, and of these, 13 (52%) had not progressed on larotrectinib at the time of analysis, thus potentially overestimating the proportion of patients with a GMI of <1.33. Therefore, in an exploratory analysis, we used a non-parametric method with ranking of the paired PFS/TTP times, in which censored PFS times were handled by a mid-rank computation [7]. This estimated the probability of attaining a GMI of ≥1.33 for all patients, including PFS-censored patients, to be 0.75 (95% CI, 0.65–0.85). A GMI waterfall plot demonstrated that patients across various tumor types met the GMI threshold of ≥1.33 (Figure 2A). In a parallel analysis, the Kaplan–Meier estimate indicated that the median GMI was 6.46 (95% CI, 4.67–NE; Figure 2B).

Among patients with metastatic disease (*n* = 53), arithmetic median GMI was 2.87 (range 0.01–48.75); 35 patients (66%) had a GMI of ≥1.33, 32 patients (60%) had a GMI of ≥2, and 16 patients (30%) had a GMI of ≥5. The probability of attaining a GMI of ≥1.33 for these patients was 0.68 (95% CI, 0.55–0.80). GMI in patients with metastatic disease was similar in subgroups divided by age, ECOG performance status, *NTRK* gene fusion, and number of prior lines of treatment (Table 2).

## 3. Discussion

Efficient trial designs are particularly important in rare malignancies for which randomized trials are less feasible. In an intra-patient comparison, individual patients serve as their own control, thus minimizing differences in non-treatment-related variables and allowing comparison of two treatments. This method also accounts for the molecular heterogeneity of tumors, particularly when a tumor-agnostic agent is being evaluated. GMI analysis was originally proposed for use in trials involving cytostatic agents and a number of phase II studies of cytotoxic anticancer treatments have implemented this approach using a GMI of ≥1.33 as a threshold for meaningful clinical activity [11,12,13,14,15]. We believe that GMI may be more broadly applied in studies investigating targeted therapies in molecular niches for rare malignancies.

The arithmetic median GMI in our analysis was 2.68, more than double the 1.33 threshold suggested in the literature to consider a drug as efficacious. Moreover, nearly two-thirds of patients had a GMI of ≥1.33 and more than one-quarter had a GMI of ≥5. These proportions may be an underestimation since >60% of patients had not progressed on larotrectinib, meaning that their PFS will increase with longer follow-up. Since these patients were not censored from the GMI analysis, we performed a non-parametric analysis adjusting for censoring, which showed a 0.75 probability of a GMI of ≥1.33 for the overall dataset. Using the Kaplan–Meier product-limit estimator, the median GMI of 6.46 is more than two times higher than the arithmetic median. Furthermore, we compared TTP on prior treatment and PFS on larotrectinib using a Kaplan–Meier analysis that censored the patients who had not progressed on larotrectinib by the data cut-off. These results demonstrated substantially longer PFS (HR, 0.220) on larotrectinib compared with TTP on the prior line of therapy, confirming the results of the GMI analysis.

One limitation of this analysis is that patients had to have received at least one prior therapy to be included in the GMI analysis; therefore, it is possible that the patients with the poorest prognoses (including those who had died on prior treatment) were excluded. Since PFS on larotrectinib includes the time from larotrectinib initiation to death from any cause, it could potentially underestimate GMI by underrepresenting all the patients that would benefit from treatment.

Another limitation of this analysis is that TTP on the most recent prior therapy was based on retrospective data collected during routine clinical care while PFS on larotrectinib was calculated from prospective clinical trial data. In addition, not all patients progressed on prior therapy; some patients experienced treatment failure (i.e., adverse event or lack of clinical benefit) and were enrolled into a larotrectinib trial due to physician decision. The retrospective TTP assessments may have been performed on different schedules (leading to potential time-assessment bias) and/or with different modalities than the standardized imaging conducted in a clinical trial. Furthermore, the date of progression on the most recent prior therapy was not known for many of the patients, so the date of progression was either the next nearest month and year of the last day of prior therapy or the start of larotrectinib. Regardless, either method would lead to an overestimate of the TTP, since the patients entered the larotrectinib trials due to progression (or failure) on prior therapy and that event would have occurred prior to the start of larotrectinib. This overestimation of the TTP would therefore result in a conservative estimate of the GMI. It should be noted that GMI has been debated since it requires strong correlation between TTP and PFS. This is based on the premise that patients who have relatively short (or long) TTP on a previous treatment are expected to have relatively short (or long) PFS on a subsequent treatment. A proposed study design using sequentially measured paired TTP/PFS data as a statistical framework to demonstrate that a strong positive correlation within an individual would require a smaller sample size and may be highly efficient [6]. However, the marked improvement in GMI seen with larotrectinib, especially as the majority of patients were free of progression at the data cut-off, together with the high ORR and long durability of disease control previously reported [4], is very promising. Larotrectinib has demonstrated clinically meaningful, tumor-agnostic efficacy and a favorable safety profile irrespective of age, *NTRK* gene, or fusion partner, showing great potential as a targeted therapy [4,5]. Another limit of the GMI assessment is the potential heterogeneity of a tumor over time that may result in variable tumor cell growth dynamics. Finally, as this was a single-arm analysis without a comparative control arm, it is possible confounding of time may have occurred.

Notably, the proportion of patients with a GMI of ≥1.33 in our analysis remains far higher than that reported in the MOSCATO 01 trial (NCT01566019), one of the largest precision medicine studies to use high-throughput molecular analysis to guide targeted therapy for patients with advanced solid tumors. In that study, GMI was >1.3 in 33% of the patients treated with a drug matched to their tumor’s molecular profile [17].

In order to explore which patients could derive more benefit from TRK inhibition, we also assessed the GMI in subgroups. The proportion of patients with a GMI of ≥1.33 did not differ according to age, performance status, or number of prior lines of therapy, although it should be noted that these subgroups had small numbers of patients.

## 4. Materials and Methods

Patients were enrolled in a phase I trial in adults (NCT02122913), a phase I/II trial in pediatric patients (SCOUT, NCT02637687), or a phase II trial in adults and adolescents (NAVIGATE, NCT02576431). Full details are described elsewhere [4].

**Figure 3:**
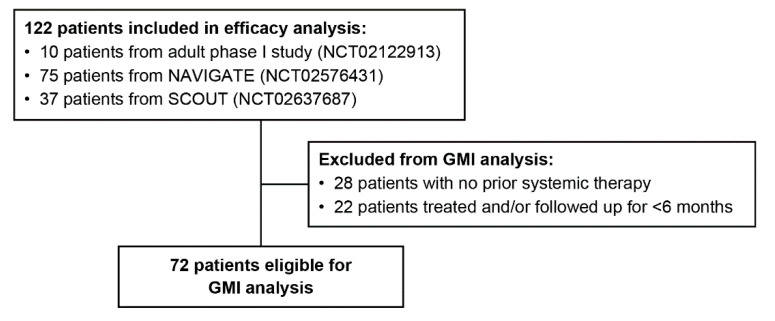
Growth modulation index (GMI) analysis set.

A retrospective, exploratory analysis of GMI was conducted in patients with non-central nervous system solid primary tumors with an *NTRK* gene fusion and measurable disease who had received at least one prior line of systemic treatment for locally advanced or metastatic disease and had been treated with larotrectinib and followed up for at least 6 months (Figure 3). Tumor types represented in the efficacy analysis were soft tissue sarcoma (23%), salivary gland (16%) thyroid (15%), infantile fibrosarcoma (15%), lung (9%), melanoma (6%), colon (5%), gastrointestinal stromal tumor (3%), bone sarcoma (2%), breast (2%), cholangiocarcinoma (2%), appendix (1%), congenital mesoblastic nephroma (1%), pancreatic (1%), and cancer of unknown primary (1%).

GMI was defined as the ratio of PFS on larotrectinib to TTP on the most recent prior line of therapy on which the patient had just progressed [8]. PFS was defined as the time from larotrectinib initiation to radiological progression (Response Evaluation Criteria in Solid Tumors (RECIST) v1.1 per independent review committee (IRC)), clinical progression, or death from any cause. Censoring rules were applied for PFS as described in the statistical analysis plan. TTP was defined as the time from the start of the last prior treatment to radiological progression (RECIST v1.1), treatment failure, or clinical progression. A subset of patients did not progress on prior therapy but had been deemed to have failed prior therapy due to a stable disease, an adverse event, or a patient and/or physician decision. Of the 72 patients involved in the study, 24 patients had failed on prior therapy. For the purposes of these analyses, treatment failure events were treated as treatment progression events. For purposes of TTP calculation, incomplete or missing dates of progression on last prior treatment were imputed conservatively to the next nearest month (for missing days), year of end of prior treatment (for missing months), or the start of larotrectinib treatment (for missing dates). Waterfall plots were used to illustrate the GMI of individual patients. The Kaplan–Meier method was used to estimate GMI as well as the PFS and TTP curves. For the PFS Kaplan–Meier curve, patients who had not progressed by the data cut-off were censored. Subgroup analyses were performed according to age, sex, ECOG performance status, tumor type, and lines of prior therapy. Statistical analyses were performed using SAS^®^ version 9.2 (SAS Institute Inc., Cary, NC, USA). The date of data cut-off was July 30, 2018.

All studies were done in accordance with the standard of good clinical practice, the principles expressed in the Declaration of Helsinki, and all applicable country and local regulations. Protocols were approved by an institutional review board or independent ethics committee at each investigative site. All patients (or parents or guardians of minor patients) provided written informed consent before the initiation of any study-related procedures.

## 5. Conclusions

The use of growth modulation indices in the assessment of investigational therapies for rare malignancies allows for the generation of comparative data by using patients as their own control, when the low number of patients available for recruitment often limits trial designs to single-arm studies. Moreover, GMI involves measures of tumor progression, which may be more relevant as endpoints than tumor response outcomes in the study of novel targeted therapies. This intra-patient comparative analysis suggests that TRK-specific inhibition with larotrectinib improves PFS in patients with TRK fusion cancer compared with prior therapies.

## Figures and Tables

**Table 1:** Patient baseline characteristics.

Characteristic	All Patients *n* = 72	Patients with Metastatic Disease *n* = 53
**Sex, n (%)**		
Male	36 (50)	22 (42)
Female	36 (50)	31 (58)
**Age, years, n (%)**		
<5	10 (14)	1 (2)
5 to <18	11 (15)	10 (19)
≥18	51 (71)	42 (79)
**ECOG performance status, n (%)**		
0	35 (49)	22 (42)
1	28 (39)	23 (43)
2	9 (13)	8 (15)
**Disease setting, n (%)**		
Locally advanced	19 (26)	0
Metastatic	53 (74)	53 (100)
**Tumor type, n (%)**		
STS: Infantile fibrosarcoma	10 (14)	3 (6)
STS: Other	16 (22)	12 (23)
Salivary gland	9 (13)	4 (8)
Lung cancer	7 (10)	7 (13)
Melanoma	7 (10)	5 (9)
Thyroid cancer	7 (10)	6 (11)
Colon cancer	6 (8)	6 (11)
Gastrointestinal stromal tumor	4 (6)	4 (8)
Cholangiocarcinoma	2 (3)	2 (4)
Appendix cancer	1 (1)	1 (2)
Bone sarcoma	1 (1)	1 (2)
Breast cancer	1 (1)	1 (2)
Pancreatic cancer	1 (1)	1 (2)
**Prior lines of treatment for advanced disease, n (%)**		
0	0	0
1	26 (36)	13 (25)
2	20 (28)	16 (30)
≥3	26 (36)	24 (45)
***NTRK*** **gene fusion, n (%)**		
*NTRK1*	35 (49)	29 (55)
*NTRK2*	2 (3)	2 (4)
*NTRK3*	35 (49)	22 (42)

Abbreviations: ECOG, Eastern Cooperative Oncology Group; *NTRK*, neurotrophic tyrosine receptor kinase; STS, soft tissue sarcoma.

**Table 2:** Growth modulation index by patient subgroup for patients with metastatic disease (*n* = 53).

Subgroup	GMI, n (% of Each Subgroup)		
	<1	1–1.33	≥1.33
**Sex**			
Male (*n* = 22)	4 (18)	2 (9)	16 (73)
Female (*n* = 31)	11 (35)	1 (3)	19 (61)
**Age group**			
Adult (≥18 years) (*n* = 42)	14 (33)	2 (5)	26 (62)
Pediatric (<18 years) (*n* = 11)	1 (9)	1 (9)	9 (82)
**ECOG** **performance status**			
0 (*n* = 22)	5 (23)	2 (9)	15 (68)
1 (*n* = 23)	9 (39)	1 (4)	13 (57)
2 (*n* = 8)	1 (13)	0	7 (88)
***NTRK*** **gene**			
*NTRK1* (*n* = 29)	8 (28)	3 (10)	18 (62)
*NTRK2* (*n* = 2)	1 (50)	0	1 (50)
*NTRK3* (*n* = 22)	6 (27)	0	16 (73)
**Lines of prior therapy**			
1 (*n* = 13)	2 (15)	0	11 (85)
2 (*n* = 16)	7 (44)	2 (13)	7 (44)
≥3 (*n* = 24)	6 (25)	1 (4)	17 (71)

Abbreviations: ECOG, Eastern Cooperative Oncology Group; GMI, growth modulation index; *NTRK*, neurotrophic tyrosine receptor kinase.

## References

[1] Vaishnavi A., Le A.T., Doebele R.C. (2015). Trking Down an Old Oncogene in a New Era of Targeted Therapy. Cancer Discov..

[2] Bayer A.G. Vitrakvi Summary of Product Characteristics. https://www.ema.europa.eu/en/documents/product-information/vitrakvi-epar-product-information_en.pdf.

[3] Bayer HealthCare Pharmaceuticals Inc Vitrakvi Prescribing Information. http://labeling.bayerhealthcare.com/html/products/pi/vitrakvi_PI.pdf.

[4] Hong D.S., DuBois S.G., Kummar S., Farago A.F., Albert C.M., Rohrberg K.S., van Tilburg C.M., Nagasubramanian R., Berlin J.D., Federman N. (2020). Larotrectinib in Patients with Trk Fusion-Positive Solid Tumours: A Pooled Analysis of Three Phase 1/2 Clinical Trials. Lancet Oncol..

[5] Federman N., McDermott R. (2019). Larotrectinib, a Highly Selective Tropomyosin Receptor Kinase (Trk) Inhibitor for the Treatment of Trk Fusion Cancer. Expert Rev. Clin. Pharmacol..

[6] Mick R., Crowley J.J., Carroll R.J. (2000). Phase II Clinical Trial Design for Noncytotoxic Anticancer Agents for Which Time to Disease Progression Is the Primary Endpoint. Control. Clin. Trials.

[7] Kovalchik S., Mietlowski W. (2011). Statistical Methods for a Phase II Oncology Trial with a Growth Modulation Index (GMI) Endpoint. Contemp. Clin. Trials.

[8] Von Hoff D.D. (1998). There Are No Bad Anticancer Agents, Only Bad Clinical Trial Designs--Twenty-First Richard and Hinda Rosenthal Foundation Award Lecture. Clin. Cancer Res..

[9] European Medicines Agency Guideline on the Evaluation of Anticancer Medicinal Products in Man. https://www.ema.europa.eu/en/documents/scientific-guideline/guideline-evaluation-anticancer-medicinal-products-man-revision-5_en.pdf.

[10] Bonetti A., Zaninelli M., Leone R., Franceschi T., Fraccon A.P., Pasini F., Sabbioni R., Cetto G.L., Sich D., Brienza S. (2001). Use of the Ratio of Time to Progression Following First- and Second-Line Therapy to Document the Activity of the Combination of Oxaliplatin with 5-Fluorouracil in the Treatment of Colorectal Carcinoma. Ann. Oncol..

[11] Comella P., Casaretti R., Crucitta E., De Vita F., Palmeri S., Avallone A., Orditura M., De Lucia L., Del Prete S., Catalano G. (2002). Oxaliplatin Plus Raltitrexed and Leucovorin-Modulated 5-Fluorouracil i.v. Bolus: A Salvage Regimen for Colorectal Cancer Patients. Br. J. Cancer.

[12] Cousin S., Blay J.Y., Bertucci F., Isambert N., Italiano A., Bompas E., Ray-Coquard I., Perrot D., Chaix M., Bui-Nguyen B. (2013). Correlation between Overall Survival and Growth Modulation Index in Pre-Treated Sarcoma Patients: A Study from the French Sarcoma Group. Ann. Oncol..

[13] Penel N., Demetri G.D., Blay J.Y., Cousin S., Maki R.G., Chawla S.P., Judson I., von Mehren M., Schoffski P., Verweij J. (2013). Growth Modulation Index as Metric of Clinical Benefit Assessment among Advanced Soft Tissue Sarcoma Patients Receiving Trabectedin as a Salvage Therapy. Ann. Oncol..

[14] Bachet J.B., Mitry E., Lievre A., Lepere C., Vaillant J.N., Declety G., Parlier H., Emile J.F., Julie C., Rougier P. (2009). Second- and Third-Line Chemotherapy in Patients with Metastatic Pancreatic Adenocarcinoma: Feasibility and Potential Benefits in a Retrospective Series of 117 Patients. Gastroenterol. Clin. Biol..

[15] Demetri G.D., Chawla S.P., von Mehren M., Ritch P., Baker L.H., Blay J.Y., Hande K.R., Keohan M.L., Samuels B.L., Schuetze S. (2009). Efficacy and Safety of Trabectedin in Patients with Advanced or Metastatic Liposarcoma or Leiomyosarcoma after Failure of Prior Anthracyclines and Ifosfamide: Results of a Randomized Phase II Study of Two Different Schedules. J. Clin. Oncol..

[16] Austin P.C. (2014). The Use of Propensity Score Methods with Survival or Time-to-Event Outcomes: Reporting Measures of Effect Similar to Those Used in Randomized Experiments. Stat. Med..

[17] Massard C., Michiels S., Ferte C., Le Deley M.C., Lacroix L., Hollebecque A., Verlingue L., Ileana E., Rosellini S., Ammari S. (2017). High-Throughput Genomics and Clinical Outcome in Hard-to-Treat Advanced Cancers: Results of the Moscato 01 Trial. Cancer Discov..

